# An examination of studies related to the sport of curling: a scoping review

**DOI:** 10.3389/fspor.2024.1291241

**Published:** 2024-02-13

**Authors:** Emily Zacharias, Nicole Robak, Steven Passmore

**Affiliations:** Perceptual Motor Behaviour Laboratory, Faculty of Kinesiology & Recreation Management, University of Manitoba, Winnipeg, MB, Canada

**Keywords:** sport of curling, wheelchair curling, curl mechanism, sport technology, quantitative measures, curling strategy

## Abstract

**Introduction:**

There has been growth in research in the sport of curling over the past few decades. The need for a scoping review is warranted. This study's purpose was to identify and synthesize research evidence regarding quantitative variables for a series of components within the sport of curling.

**Methods:**

A scoping review of studies published and established within four databases was performed. One independent reviewer selected studies based on a systematic procedure. Inclusion criteria for studies were: (1) interventions that focused on the sport of curling; (2) quantitative in nature; (3) written in English; and (4) published within a peer-reviewed journal, a conference presentation, or a published thesis.

**Results:**

Searching identified 8,467 articles and 94 met the inclusion criteria. Data were organized and synthesized based on the devised research variables from the sport of curling: The curl mechanism of the curling stone; the impact of sweeping on stone trajectory; curling delivery mechanics; wheelchair curling; technology analysis; strategy and tactics; psychological factors; injury occurrences; facility and arena infrastructure; and assessment of curling training and ability. The findings confirm the strong knowledge base that exists across game variables and unveil controversy between the underlying physics that produces curl, as well as the mechanisms of sweeping responsible for manipulating the stone trajectory.

**Conclusions:**

Knowledge derived from this review can assist researchers, coaches, and curlers in addressing the specific variables of the sport that influence stone trajectory and game results. Such awareness will expose gaps in the current understanding and foster new research endeavors to further the knowledge of the sport.

## Introduction

Curling is a target-directed winter sport with origins that date back to Scotland in 1530 (1). Today, the modern game is played with granite rocks on an indoor 42 m pebbled ice surface and is increasingly advancing in its rules and geographic popularity and is played at both a recreational and professional level (2). It has evolved into an Olympic and Paralympic sport with Women's, Men's, Mixed Doubles, and Mixed Wheelchair divisions (3). The traditional version of the sport consists of two teams, made up of four players per team, each throwing eight stones per end. The team with the advantage of throwing the last rock of the end has the “hammer”. By having the “hammer” the likelihood of winning an end is potentially increased. It is increased because the thrower can either knock out an opponent's stone or position their stone closer to the center pin. Each team member throws two consecutive stones per end alternating with the opposing team, translating the rock from the hack to the far house by releasing it at the near hog line (2). The rocks thrown with a linear and angular velocity travel down the ice while curling laterally across the sheet depending on the rotation applied to the handle. Team members not throwing act as sweepers, brushing in front of the rock in an attempt to influence the motion of the rock. The winning team of the end collect points at the completion of each end by having the stone closest to the center pin, and they receive additional points for each of their stones that rests in the house closer to the pin than the nearest opponent stone. The goal is to have the most points after the final end (3).

This sport, commonly known as “chess on ice”, is a combination of skill and tradition, with a unique set of physical demands (2). It comprises many variables including strategy and tactics, delivery biomechanics, sweeping techniques, emotional control, and team cohesion. The sport is highly variable, dependent on the state of the ice, the initial stone velocity, the ability to deliver the rock on target, and the number of rock rotations. Further, variability exists in stone trajectory due to the influence of sweepers brushing at varying speeds and positions (2). Technological advancements are constantly evolving the game while scientific data and field experience play an essential role in accumulating knowledge for each variable of the game, to allow for progression in competition and athleticism (4). The sport has many quantitative variables that determine the motion of the curling rock and the scoreboard result; thus, it appeared vital to review and summarize this published data. Through investigation, no comprehensive scoping review currently exists to examine all quantitative research conducted on the sport.

The purpose of this scoping review is (1) to consider all quantitative research that has been conducted in the sport of curling in order to understand the depth of our current knowledge in this field, (2) to establish the gaps in our understanding of the sport of curling, and (3) to determine future directions of study within the sport to inform athletes, coaches, as well as researchers on how to optimize play.

## Methods

A scoping review of the literature was appropriate to meet the objectives of this study and answer the research question: what variables have been collected in curling studies to date, and are they meaningful to the multifaceted sport or prioritized as a scientific variable only?

The protocol was developed using the scoping review methodological framework adopted from Arksey and O'Malley (5). The draft protocol for the review was analyzed by research colleagues and implemented. The protocol consisted of a series of five stages, details of the search strategy and steps of the review process included:

(1) Identifying and collecting relevant studies: literature searches were conducted across four electronic bibliographic databases which include: PubMed, SPORTDiscus, Scopus, and Google Scholar. An initial search, using the search term “Curling” was conducted using Harzing's Publish or Perish software (6). This search established salient parameters and eight key search terms to carry out additional searches across the four databases. Those eight key terms included: (i) curling rock; (ii) sweeping effect in curling; (iii) curling team dynamics; (iv) curling delivery; (v) curling brush; (vi) cognitive imagery in curling; (vii) curling slide; and (viii) motion of curling rock. The collected literature was then screened for relevance in the sport of curling. After removing duplicates, studies were assessed for eligibility. Reference lists of eligible studies were further screened for additional, relevant studies.
(2) Selecting of studies: inclusion and exclusion criteria were established to filter and guide searches for relevant literature. In order to be included, literature from searches had to meet four inclusion criteria: (i) be from a peer-reviewed journal, a conference presentation, or a published thesis; (ii) published in the English language; (iii) include documented interventions or analysis in the sport of curling; and (iv) be quantitative in nature and/or describe quantitative research variables (mathematical modeling or statistical comparison). The literature was not restricted in time frame, study population, geographical publication, or type/design of journal article. Collected literature that did not meet all criteria was excluded, however, conflicting literature were analyzed by two colleagues to reach consensus for inclusion. By applying the eligibility criteria, two reviewers screened the articles for selection. Blinding was applied at this stage to ensure no bias between reviewers in the selection process. All conflicts between the two reviewers, generated through screening, were discussed to reach consensus. When conflict remained, the opinion of a third reviewer was sought to reach consensus. Initially, articles were selected from the title and abstract screening. A second, more in-depth selection, was then conducted through full-text screening. June 1, 2021 was the last date that the search was executed.
(3) Charting the data: once included articles were selected, data was extracted and charted according to author, title, journal, publication year, geographical location, purpose, sample size and type, methodology, intervention type, outcomes, key findings, and barriers. One author extracted and grouped the data, and another validated the data to ensure accuracy. Data were organized and grouped into subtopics according to the identified study purposes (i) the curl mechanism of the curling stone; (ii) the impact of sweeping on stone trajectory; (iii) curling delivery mechanics; (iv) wheelchair mechanics; (v) technology and tools; (vi) strategic/tactical analysis; (vii) psychological aspects; (viii) injury occurrences; (ix) impact of facility infrastructure on ice surfaces; and (x) assessment of curling training and ability.
(4) Summarizing and synthesis of the results: authors collectively compared and discussed the charted data. Descriptive statistics were performed to characterize the research literature and to identify the breadth and gaps. Trends across geographic location and decades of publication of included studies were evaluated. The study results were examined and discussed within each curling variable, to determine trends and commonalities. Barriers and gaps were identified within the literature to suggest future areas of study. Consensus between all three authors regarding the key items of information generated from the review was reached.

In addition to the scoping review methodological framework proposed by Arksey and O'Malley (5), we followed the PRISMA Extension for Scoping Reviews (PRISMA-ScR) checklist (7). No risk of bias assessment, summary measures, or additional analyses were conducted in this scoping review in accordance with the PRISMA-ScR (7). No formal review protocol exists.

## Results

**Figure 1:**
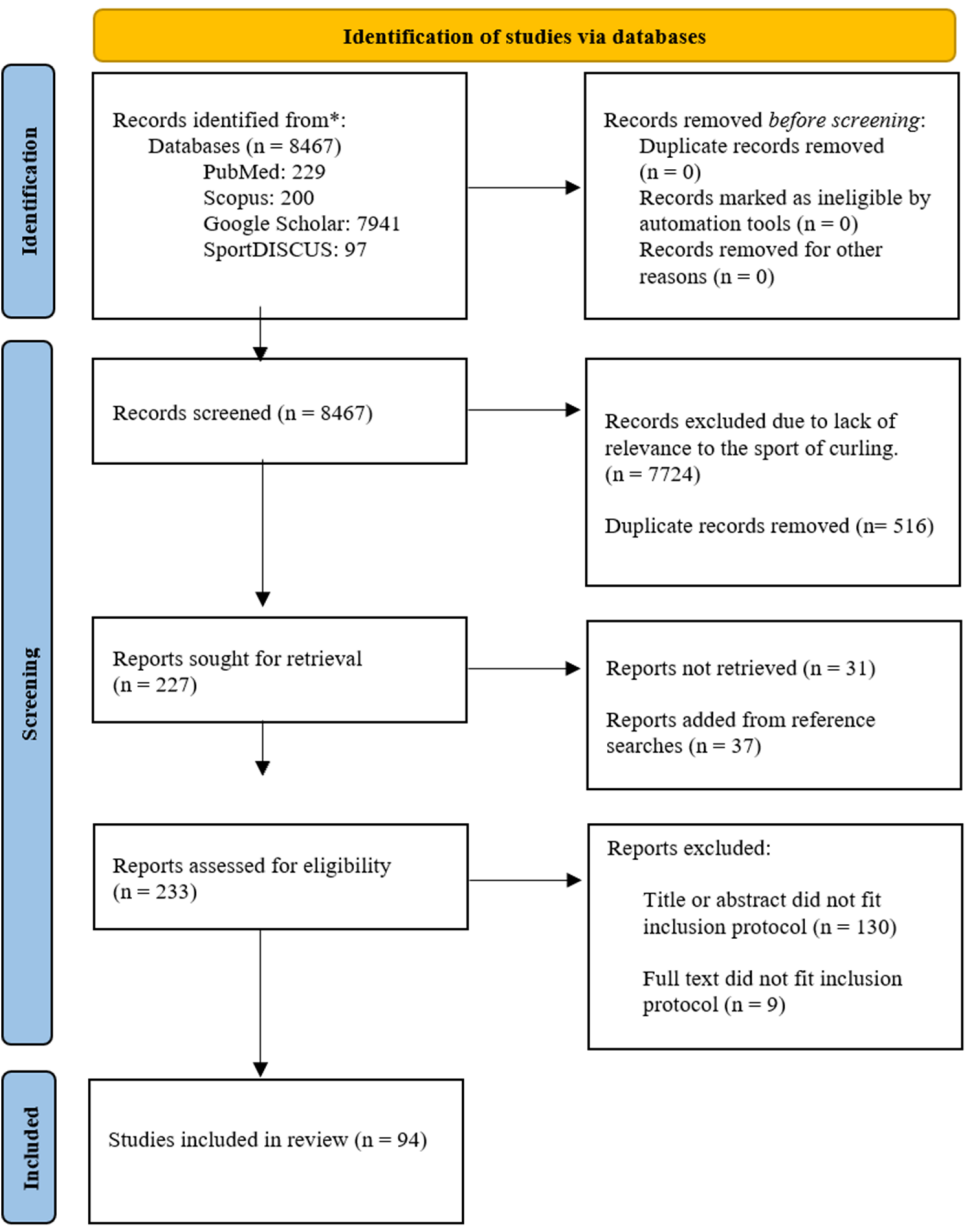
PRISMA 2020 flow diagram: overview of the study identification and selection process.

The initial search from the electronic databases using Harzing's Publish or Perish software yielded 1,367 results (PubMed: 167, Scopus: 200, Google Scholar: 1,000) (Figure 1). The thorough searches, using eight predetermined search terms, that followed the initial search, yielded 7,100 results (PubMed: 62, SPORTDiscus: 97, Google Scholar: 6,941, Scopus: 0). Therefore, a total of 8,467 results were retrieved through database searches. These records were screened to eliminate studies lacking relevance to the sport of curling, narrowing the results to 743. Duplicates were then filtered out leaving 227 records. The reference lists were screened for additional, relevant studies. Thirty-seven studies were added from reference searches to the existing 227, leaving 264 studies remaining. We obtained full-text access to 233 of these and after the initial screening of titles and abstracts 103 fit the inclusion protocol and remained. An additional nine articles were excluded after full-text assessment due to not fitting inclusion criteria (iv). Therefore, a total of 94 articles were included in the final data extraction, quality appraisal, and review in order to evaluate the quantitative variables in curling.

### Study characteristics

**Figure 2:**
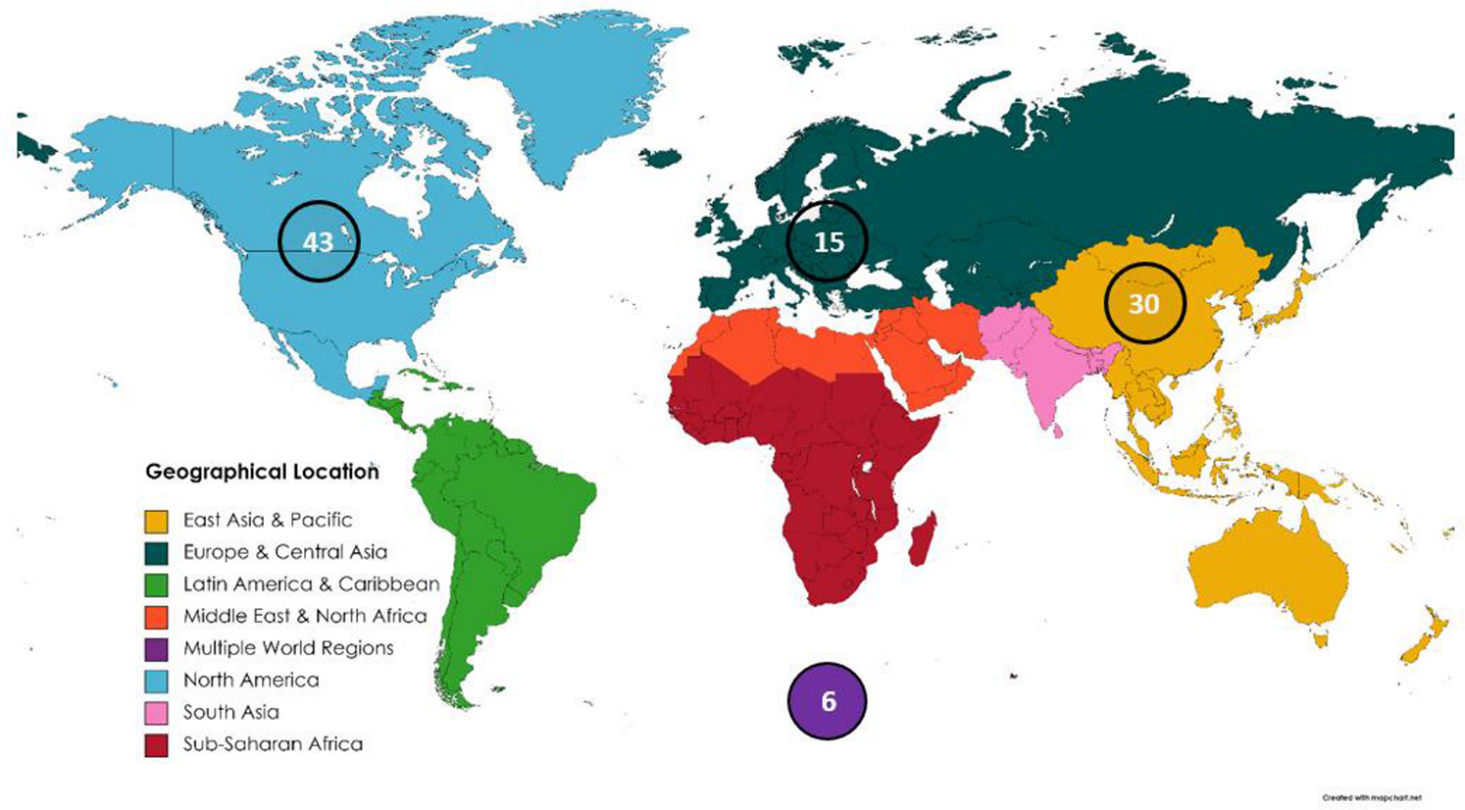
Number of included studies by geographical location.

Geographical location: when analyzing the geographic scope of the studies, two world regions accounted for a large sum (Figure 2). Forty-three of the ninety-four included studies (46%) have an origin in North America, with 36 of these studies emerging from Canada alone. A bias toward English speaking countries is possible due to one of the study inclusion criteria specifying that articles were to be written in English. Another possible bias is that curling is a sport that requires an indoor ice facility that may be cost prohibitive in some regions of the world. East Asia contributed 32% (30/94) of the studies respectively. Within this region, the studies are divided among three major countries, with Japan leading at 18, and then Korea and China close behind with eight and four. Up to nine European countries are also represented in the list of included studies ranging across the Nordic, Southern, and Western parts, which make up 16% (15/94). The remaining 6% (6/94) of studies are combinations of multiple world regions such as Korea and Germany collaborating or Canada collaborating with a variety of countries (Sweden, Scotland, Netherlands, or the United States of America).

**Figure 3:**
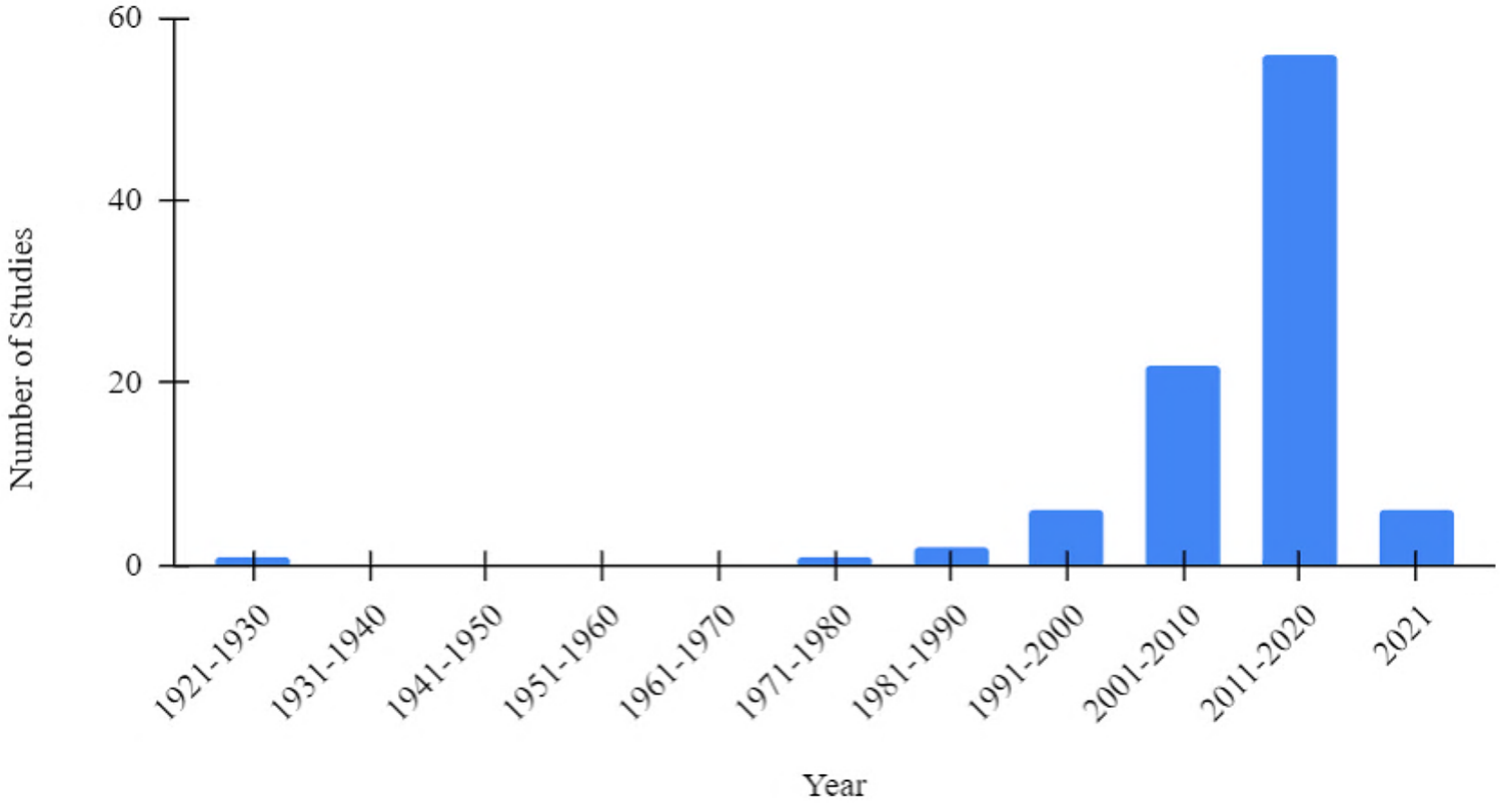
Number of included studies by decade (1921–2021). The publications in 2021 only include search's since June 1 of 2021.

Decades of Publication: There has been a substantial increase in the number of articles published on the sport of curling in recent decades (Figure 3). The sport of curling was recognized as an Olympic sport in 1998 and the sport of Wheelchair curling as a Paralympic sport in 2002 (1). In 2007 the sport grew in popularity across Asia with the first World Women's Curling Championship taking place in Aomori, Japan (1). Among the included studies, 60% (*n* = 56) fell between 2011 and 2020, more than doubling the 23% (*n* = 22) of studies published between 2001 and 2010. From the months of January until July of 2021, an additional six studies (6%) had already been published. Conversely, only 9 (10%) studies were published between 1970 and 2000, and only one included study (1%) was published before 1970, in 1924.

### Themes

Through the outcomes assessed in the literature, multiple themes emerged. These themes were identified and categorized by one researcher with curling expertise and studies were grouped into 10 categories inspired by different domains in the sport of curling. Most studies evaluated one specific theme within the sport while acknowledging others to a lesser degree, however, some overlap of themes emerged in studies. Table 1 groups all studies by theme, variable, citation, and geographical region.

**Table 1:** Summary of all themes, the variable(s) assessed in each theme, and the studies that assessed the variable(s).

Theme	Sub-theme	Variable	Author(s)	Country
Assessment of curling training and ability		Off-ice athletic ability and physiological tests	Kivi et al. (8)	Canada
		On-ice technical, strategic, and shot-making skills	McNeil (9)	Canada
			Kivi et al. (8)	Canada
			Zhang et al. (10)	China
Curling arena infrastructure		Construction and tranformation of non-curling fields into curling ice rinks	Li et al. (11)	China
	** **	Injury according to force vector analysis of delivery positions	Robertson et al. (12)	UK
	** **		Reeser and Berg (13)	USA
Injury analysis in curling		Injuries according to demographics, site of injury, type of injury, and curling-specific conditions	Berry et al. (14)	USA
		Psychological performance of coaches	Paquette (15)	Canada
		Psychological performance of older adults	Stone et al. (16)	Canada
			Stewart (17)	Canada
			Pezer and Brown (18)	Canada
			Lizmore (19)	Canada
			Kim et al. (20)	Korea
Psychological aspects of curling		Psychological skill training	Kim et al. (21)	Korea
			Yamamoto et al. (22)	Japan
Strategic/tactical factors		Using AI-based programs to propose strategic learning methods	Han et al. (23)	China
		Assessing hammer scenarios	Willoughby and Kostuk (24)	Canada, USA
			Willoughby and Kostuk (25)	Canada, USA
			Ahmad et al. (26)	Canada
			Park and Lee (27)	Korea
			Ahmad (28)	Canada
		Studying game information using the AI program iCE	Otani et al. (29)	Japan
			Otani et al. (30)	Japan
			Masui et al. (31)	Japan
			Masui et al. (32)	Japan
		Game-tree searches of optimal shot selection via jiritsu-kun programming	Yamamoto et al. (22)	Japan
		Continuous action spaces via the Markov process	Ohti and Tanaka (33)	Japan
			Kostuk et al. (34)	Canada, Netherlands, USA
		Deep reinforcement learning framework	Yamamoto et al. (22)	Japan
			Won et al. (35)	Germany, Korea
			Lee et al. (36)	Korea
		Neural networking of curling strategy from continuous action spaces using Monte Carlo Tree Search and kernel regression algorithms	Yee et al. (37)	Canada
			Lee et al. (36)	Korea
			Han et al. (23)	China
		Intelligent curling elicitation (iCE)	Masui et al. (31)	Japan
			Masui et al. (32)	Japan
			Masui et al. (38)	Japan
		Artificial infrastructure for position measurement and trajectory of curling stone	Takahashi et al. (39)	Japan
			Lee et al. (40)	Korea
			Ravanbod (41)	Canada
			Takegawa (42)	Japan
			Ohti and Tanaka (33)	Japan
		Digital curling system	Ito and Kitasei (43)	Japan
		Musculoskeletal analysis of upper body kinematics and release velocity	Tanaka et al. (44)	Japan
		AI vs. human curling matches	Won et al. (35)	Germany, Korea
			Choi et al. (45)	Korea
			Aannevik and Robertsen (46)	Norway
			Won et al. (47)	Germany, Korea
			Won et al. (35)	Germany, Korea
			Tanaka et al. (44)	Japan
			Takahashi et al. (39)	Japan
			Ravanbod (41)	Canada
			Kawamura et al. (48)	Japan
Integrated technology and artificial intelligence in curling		Design and implementation of robotic machines	Gwon et al. (49)	Korea
			Choi et al. (45)	Korea
			Aannevik and Robertsen (46)	Norway
		Psychological skill training	Kim et al. (20)	Korea
		Musculoskeletal modeling and body composition measurements	Laschowski and McPhee (50)	Canada
			Laschowski (51)	Canada
		Trunk and motor control stability	Laschowski and McPhee (50)	Canada
			Laschowski (51)	Canada
			Laschowski et al. (52)	Canada
			Herzog et al. (53)	Switzerland
			Laschowski et al. (52)	Canada
Wheelchair curling		Kinematic trajectories	Laschowski and McPhee (50)	Canada
			Laschowski (51)	Canada
		Cross-gender comparison of movement and hip flexibility	Kraemer (54)	USA
		Comparison of elite and sub-elite curlers	Shank and Lajoie (55)	Canada
			Kivi and Auld (56)	Canada
			Pojskic et al. (57)	Canada, Sweden
			Bernier et al. (58)	USA
		Force generation and weight control	Harms (59)	USA
			Kivi and Auld (56)	Canada
		Delivery variables according to movement phases	Shank and Lajoie (55)	Canada
			Kraemer (54)	USA
			Kivi and Auld (60)	Canada
Curling delivery mechanics		Biomechanical force analysis	Robertson et al. (12)	UK
		Flexibility, balance, and core strength	Pojskic et al. (57)	Canada, Sweden
			Bernier et al. (58)	USA
		Non-contact optical profiler and optical microscope to capture sweep data	Balsdon and Wood (61)	Canada
		Camera to capture sweep data	Kivi et al. (62)	Canada
		Electromyography to capture sweep data	Kim et al. (4)	Korea
		Inertial measurement unit (IMU) to capture sweep data	Kim et al. (4)	Korea
			Dzikowski et al. (63)	Poland
			Yanagi et al. (64)	Japan
			Marmo et al. (65)	UK
	Sweep measurement equipment	Sweep ergometer to capture sweep data	Buckingham et al. (66)	UK
			Buckingham (67)	UK
		Scratch mechanism of sweeping	Balsdon and Wood (61)	Canada
	Sweep theories	Thermomechanical theory of sweeping	Yanagi et al. (68)	Japan
			Yanagi et al. (64)	Japan
			Marmo et al. (65)	UK
			Lawson and Rave (69)	USA
		Assessing different cases of sweeping	Lawson and Rave (69)	USA
		Observing the topography and thermodynamics of ice surface when comparing different broom head material	Yanagi et al. (68)	Japan
			Marmo et al. (65)	UK
			Balsdon and Wood (61)	Canada
Impact of sweeping on the curl mechanism	Sweep variables	Development of instruments to gauge quantitative sweeping effects	Yanagi et al. (64)	Japan
			Buckingham et al. (66)	UK
			Buckingham (67)	UK
		Effects of broom acceleration vs. force production on stone trajectory and heat transfer	Yanagi et al. (70)	Japan
			Bradley (2)	Ireland
		Rates of change in stone displacement according to sweeping	Yanagi et al. (70)	Japan
			Kim et al. (4)	Korea
		Stroke rate comparison between genders	Kivi et al. (62)	Canada
			Kim et al. (4)	Korea
		Muscle activity and force output	Kim et al. (4)	Korea
		Thermodynamic analysis of ice friction	Lozowski et al. (71)	Canada
		Surface profiles and cross-scratches of ice sheets	Kameda et al. (72)	Japan
			Honkanen et al. (73)	Finland
		Friction coefficients and the function of forces acting on the stone	Penner (74)	Canada
			Nyberg et al. (75)	Sweden
			Lozowski et al. (71)	Canada
		Torque due to dry and wet friction	Shegelski et al. (76)	Canada
			Shegelski et al. (77)	Canada
		Translational and rotational stone speeds	Shegelski and Niebergall (78)	Canada
		The stone’s kinetic energy	Shegelski and Lozowski (79)	Canada
		Longitudinal and rotational deceleration of the stone	Lozowski et al. (80)	Canada
		The relationship between the linear and angular velocity of the stone	Denny (81)	Canada, Scotland
	Numerical models	Dynamic motion and trajectory of a curling stone	Shegelski (82, 83)	Canada
			Shegelski (84)	Canada
			Shegelski and Lozowski (85)	Canada
			Maeno (86)	Japan
			Honkanen et al. (73)	Finland
		Split friction/granular models	Ziegler (87)	Germany
			Shegelski et al. (76)	Canada
		Pivot-slide model	Shegelski and Lozowski (85)	Canada
			Shegelski and Lozowski (79)	Canada
			Mancini and de Schoulepnikoff (88)	Germany
			Nyberg et al. (75)	Sweden
		Scratch guide model	Shegelski and Lozowski (89)	Canada
			Penner (90)	Canada
			Honkanen et al. (73)	Finland
		Water layer model	Summers and Montgomery (91)	Canada
			Shegelski et al. (77)	Canada
			Shegelski and Niebergall (78)	Canada
			Shegelski et al. (76)	Canada
			Maeno (92)	Japan
			Jensen and Shegelski (93)	Canada
		Pressure difference model	Maeno (92)	Japan
			Johnston (94)	Canada
			Harrington (95)	Canada
Curl mechanism/physics of rock displacement	Physical theories	Snowplow model	Maeno (92)	Japan
			Denny (81)	Canada, Scotland
		Evaporation abrasion model	Maeno (92)	Japan
			Maeno (86)	Japan
			Maeno (96)	Spain
		Asymmetrical models	Penner (74)	Canada
			Nyberg et al. (97)	Sweden
			Nyberg et al. (75)	Sweden
			Denny (81)	Canada, Scotland
			Denny (98)	Scotland
			Bradley (2)	Ireland

From the included literature: (1) the curl mechanism of the curling stone; (2) the impact of sweeping; (3) curling delivery biomechanics; (4) wheelchair curling; (5) technology and tools; (6) psychological; and (7) strategic variables, all occupied the primary purpose of the greatest number of studies. Other pertinent topics included: (8) injury analysis: (9) curling facility infrastructure; and (10) assessment of curling training, which were the secondary focus of some studies and integrated into studies with another primary focus.

#### Curl mechanism/physics of rock displacement

Twenty-seven studies addressed the curl phenomenon of the granite stone on a pebbled ice surface. The main focus of these studies was to comprehend the physics-based behavior of a curling rock via theoretical mechanisms and their mathematical models.

**Figure 4:**
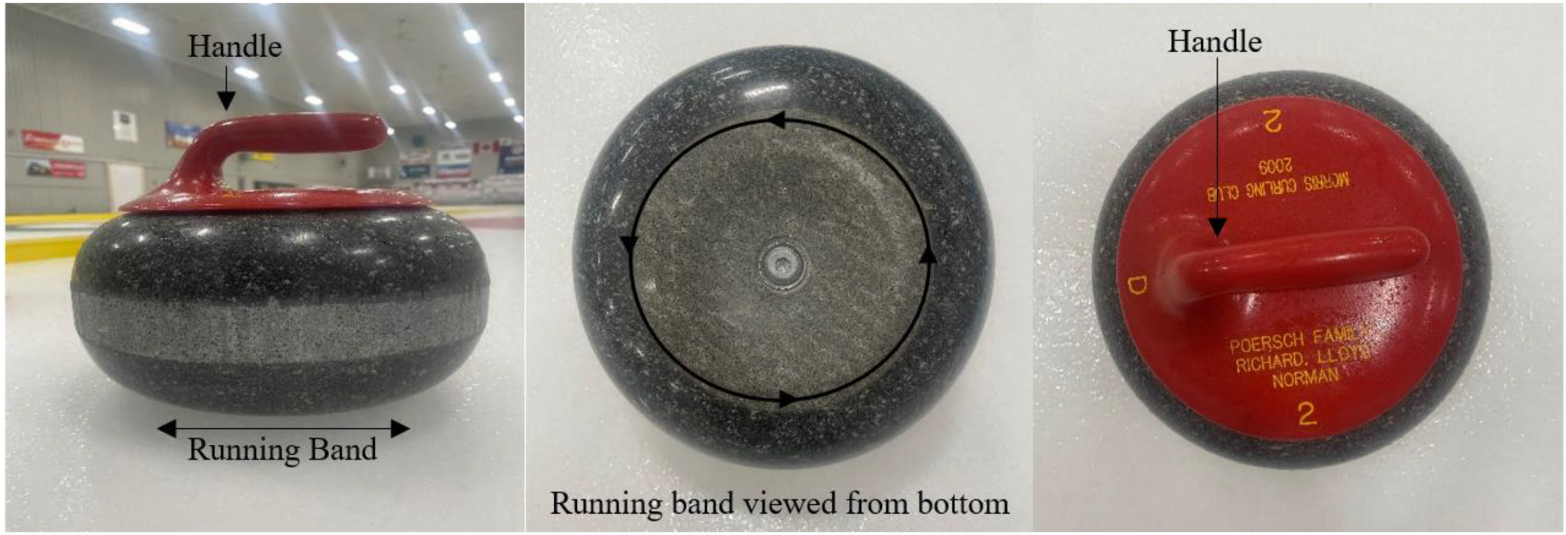
Curling stone features: front view, bottom-up view, top-down view.

Numerous physical theories were introduced as mechanisms responsible for the observed curl of a curling rock. Six of these studies assessed lateral displacement of the stone according to asymmetrical models, based on the front-back and left-right asymmetrical distribution of friction force acting on the sliding stone (2, 74, 75, 81, 97, 98). Several additional studies discussed adjacent models, sharing the same front-back asymmetrical mechanism to explain lateral curl: (i) the “evaporation abrasion model”, which considers the evaporation of pebbles as responsible for the lower friction coefficient at the front of the running band (Figure 4) (86, 92, 96); (ii) the “snowplow model”, which explains curl according to the effect of ice fragments and debris on reducing the friction coefficient (81, 92); (iii) the “pressure difference model”; the only model with statistical evidence supporting its unreliability (92, 94, 95). Further front-back asymmetrical models include the “water layer model”, in which a thin water film produced by friction heat is dragged around by the rotation of the stone, lowering the coefficient of friction of the front running band (76–78, 91–93). Four studies described the “scratch-guide model”; this theoretical framework focuses on the lateral displacement of subsequent stones as proportional to the angle of cross-scratches produced by the leading and trailing running bands of preceding stones (73, 75, 89, 90). Several studies introduced the “pivot-slide model” which describes lateral displacement as a sequence of pivots about individual ice pebbles followed by slides (79, 85, 88). Finally, two studies evaluated “split friction/granular models”, describing factors of mixed lubrication (76, 87).

Numerical models used to describe and validate the above theories were considered across studies (91, 92). Mathematical equations were presented to describe and predict: (1) the dynamic motion and trajectory of a curling stone (73, 82–86); (2) the relationship between the linear and angular velocity of the thrown stone (81); (3) the longitudinal and rotational deceleration of a stone (80); (4) the stone's kinetic energy (79); (5) the translational and rotational stone speeds (77, 78); (6) the torque due to dry and wet friction (76); (7) friction coefficients and the function of forces acting on the thrown stone (71, 74, 75, 97); (8) the surface profiles and cross-scratches of ice sheets (72, 73); and (9) thermodynamic analysis of ice friction when considering the interaction between individual ice pebbles and the stone running band (71). Two prominent mathematical analyses performed were: (1) numeric integration (74, 88), and (2) numeric differentiation (93, 96).

The common factor that all models try to account for is the coefficient of friction. Maeno (92) covers five different models that discuss the “front-back asymmetry” of frictional force in her review. Maeno does not discuss the “left-right asymmetry model” (98). Since Maeno's review, two new models were proposed bringing the total to eight. The five in Maeno's (92) review are the “Pressure Difference Model”, the “Water Layer Model”, the “Snowplow Model”, the “Evaporation Abrasion Model”, and the “Scratch Guide Model”. In order of novelty, the two newly proposed models are the “Pivot-Slide Model” (Shegelski, & Lozowski (79) and the “Split Friction Model with mixed lubrication” by Ziegler (87).

#### Impact of sweeping on the curl mechanism

Twelve studies solely explored the impact of sweeping in curling. Among these studies, variables of interest included: (1) muscle activity and force output alone and in tandem while performing the action (4); (2) stroke rate comparison between genders (4, 62); (3) rates of change in stone displacement according to sweeping (4, 70); (4) the effects of broom acceleration vs. force production on stone trajectory and heat transfer (2, 70); (5) the development of instruments to gauge quantitative sweeping effects (64, 66, 67); (7) observing the topography and thermodynamics of ice surface when comparing different broom head material (61, 65, 68); and (8) assessing different cases of sweeping (e.g., the ideal time to initiate sweeping) (69).

Two conditional theories of sweeping were assessed across studies: (1) the thermomechanical theory, outlining variables of the velocity of sweeping stroke, the downward force applied, and the pattern that is swept as having an effect on the coefficient of friction (64, 65, 68, 69); (2) the scratch mechanism of sweeping, measuring ice topography effects of sweeping; evaluating the brush head's effect on the ice surface, as well as the varying depth of scratches and the directional manipulation of the stone's trajectory (61).

Four of these studies reported using a sweep ergometer to collect force measurement and acceleration data along two axes while sweeping (64–67). Others used devices such as an inertial measurement unit (IMU) sensor attached to a broom, to measure kinematics and frequency of the sweeping activity as well as mechanical movements of a curling stone (4, 63), electromyography for muscle activity (4), cameras to collect visual data (62), and a non-contact optical profiler as well as an optical microscope to evaluate surface profiles of the ice after brush head contact (61).

#### Curling delivery mechanics

Curling delivery mechanics were the main topic of eight studies (12, 54–60). Delivery variables of flexibility, balance, and core strength were assessed in two studies (57, 58). This analysis occurred via an optotrak certus device (Northern Digital Instruments, Waterloo, ON) and a Curling-Specific Balance Test. Biomechanical force analysis was compared between flat-foot and toe sliding with toe sliding producing a larger moment arm than flat-foot sliding (12). Three of these studies assessed delivery variables according to movement phases (54, 55, 60). Phases of the slide (i.e., setup, pull-back, release, and after release) were compared in terms of attentional demands, kinematic correlations between trunk, hip, and knee movement, and directional accuracy of delivery.

Two studies considered force generation and weight control by (1) collecting data to understand the conversion of the timing of rock translation across different segments of the sheet into kick speed generated during delivery (56); (2) methods of force generation throughout the delivery includingincreased potential energy through a backswing, increased push-off from the stance leg in the hack, and earlier rock release to generate greater stone velocity (59). Comparison between elite and sub-elite curlers occurred in four studies regarding the execution and efficiency in delivery kinematics (58), movement qualities (i.e., flexibility and balance) (57, 58), timing intervals to estimate kick speed for delivery (56), and attentional demands (55). Flexibility and hip movement using hip goniometer measurements were compared across genders (54).

#### Wheelchair curling

Among the studies that investigated wheelchair curling, 50% of them assessed kinematic trajectories. These studies measured a biomechanical model of the curling delivery to quantify angular joint velocities and range of motion through the curling delivery in relation to the dynamics of stone translation (50–52). Trunk and motor control stability of the curling wheelchair athlete was assessed within four studies (50–53); this analysis concluded wheelchair curling as a beneficial method of rehabilitation for spinal cord injury (SCI) patients (53). Musculoskeletal modeling and body composition measurements effecting stone release, using dual-energy x-ray absorptiometry, were complimentary factors assessed in two studies (50, 51). The findings discuss how the distribution of fat mass in SCI athletes may be an advantage for trunk control through stone delivery and how body segment parameters influence the mass moment of inertia (50). Only one study analyzed the effects of psychological skill training on the performance of a national wheelchair team (20). A twelve-week individualized psychological skill training program was developed for each athlete. The program was informed and evaluated by in-depth interviews, numerous questionnaires (Psychological Skill Questionnaire in Sports, Questionnaire for Players' Self-Management, Korean Version of the Test of Performance Strategies, Profile of Mood States, Competitive State Anxiety Inventory [CSAI-2], and Questionnaire to Assess the Perception of Psychological Skill Training), the change in the performance outcome of the Wheelchair curling game, and EEG Inter-Hemispheric Asymmetry Index analysis. Positive psychological elements and increased competitiveness were found with improved in-game performance.

#### Integrated technology and artificial intelligence in curling

Twenty-one studies evaluated technological/artificially intelligent devices developed and implemented within the sport of curling. Thirty-eight percent (*n *= 8) of these studied the design and implementation of robotic machines across multifaceted aspects of curling (35, 39, 41, 44–49). Of the artificially intelligent (AI) curling robots, fabricated features included throwing controls to test precision and throwing accuracy, AI-based strategy simulators, vision technology to recognize the curling field and stone configuration/trajectory, and sweeping systems to deliver path planning strategies for sweeping (35, 47–49). Artificial agents were equipped with autonomous driving and friction feedback for traction control (45, 47). Of the studies that orchestrated curling matches, AI machines established a winning record while outperforming human counterparts (35, 45, 46). Other devices monitored musculoskeletal analysis of upper body kinematics and release velocity (44), as well as a digital curling system, used as a framework to compare curling strategies (33). Artificial infrastructure from four studies was evaluated with respect to their ability to monitor real-time position measurements and trajectory behavior of the curling stone, including an infrared camera (42), a prototype of a curling stone launcher (41), smart glasses (40), and a kernelized correlation filter tracker (39).

Statistical analysis and prediction of strategic curling outcomes were measured via AI algorithms in eleven studies. Three studies developed curling informatics via a digital scorebook system, intelligent Curling Elicitator (iCE) (Kitami Institute of Technology, Kitami, Japan) (31, 32, 38). Three studies focused on the neural networking of curling strategy from continuous action spaces using Monte Carlo Tree Search and kernel regression algorithms (23, 36, 37). Three of the studies implemented a deep reinforcement learning framework (22, 35, 36). Two studies evaluated continuous action spaces via the Markov process (33, 34). Single studies implemented game-tree searches of optimal shot selection via jiritsu-kun programming (22), and a server system “digital curling” to calculate turn-based curling strategy (43).

#### Strategic/tactical factors

Eleven studies considered strategic/tactical factors of the sport based on the notion of its cruciality to achieving winning outcomes. Four studies evaluated game information using the sequentially mentioned AI program iCE; a portable digital scorebook system within the novel field of curling informatics that effectively analyzes curling tactics and strategies in order to establish correlations between differences in shot accuracies and game scores (29–32).

Five studies assessed “hammer” scenarios to provide insight into tactical decisions. The importance of the “hammer” on game outcomes was considered by (1) modeling the “hammer” shot as a low-dimensional optimization problem with a continuous action space using Delaunay triangulation and sampling algorithms (26, 28); (2) applying binary logistic regression to statistics on possession of first stone or last stone per end (27); (3) appraising scenarios of being one up with the “hammer” vs. one down without, and whether to blank or take a single point (24, 25). Two studies evaluated the uncertainty of curling strategy via digital curling; AI-based programs including neural fictitious self-play (NFSP) methods (23), Kernel Regression-UCT (23), and jiritsu-kun (22) were acquired to propose strategic learning methods from expected scores distribution at the completion of ends through neural network models (22, 23).

#### Psychological aspects of curling

Seven studies focused on psychological determinants and results within the sport of curling. Psychological skill training of curling (wheelchair) athletes dominated 71% (*n *= 5) of these studies (17–21). Across the curling and wheelchair curling demographic, psychological variables of perfectionism, communication, pressure, self-management, anxiety, arousal, and interpersonal relationships were studied according to their impact on game performance (18–21). Implemented training techniques to optimize psychological state included routine training, attentional focus training, writing practice training, relation training, self-control training, positive self-talk training, and general imagery interventions, as methods of improving shot-making ability and game strategy (17, 20).

Two studies addressed psychological performance regarding other demographics (i.e., older adults and coaches) (15, 16). Both studies generated a regression model in their analysis. The psychological context within curling was evaluated using psychological skills training as a variable of coaching behavior and beliefs (15). This curling context also was assessed as a tool/leisure activity to improve older adults' psychophysical properties of functionality, balance confidence, and perceptions of aging (16). Across all seven studies, questionnaires were the most frequent form of data collection, occurring in 100% of studies (15–21). Kim and colleagues (21) utilized the Group Cohesion Questionnaire [GCQ; (99)], the Group Efficacy Questionnaire [GEQ; (100)], and the Effective Communication Questionnaire [SETECTS-2; (101)] to assess teambuilding. Kim and colleagues (20) evaluated psychological skills via the Psychological Skill Questionnaire in Sports [PSQS; (102)], the Questionnaire for Players' Self [QSF-M; (103)], the Korean Version of the Test of Performance Strategies [TOPS; (104)] the Profile of Mood States (POMS), Competitive State Anxiety Inventory (CSAI-2), and a Questionnaire to Assess the Perception of Psychological Skill Training (QAPPSK). Lizmore (19) investigated perfectionism with an abbreviated and modified version of the Sport-Multidimensional Perfectionism Scale-2 [Sport-MPS-2; (105)], and reactions to mistakes with the Sport Emotion and Cognition Questionnaire (SECQ). Paquette (15) used a revised version of the Sport-Psychology Attitudes-Revised Coaches Questionnaire [SPA-RCQ; (106)], a revised version of the Mental Skills Questionnaire [MSQ; (107)], the Coaching Competence Scale [CCS; (108)], and the Sport-Confidence Inventory [SCI; (109)]. Pezer and Brown (18) investigated personality traits by using the Will To Win Questionnaire [WTWQ; (110)]. Stewart investigated imagery ability, use, and assessment with the Movement Imagery Questionnaire-Revised [MIQ-R; (111)], and the Sport Imagery Questionnaire [SIQ; (112)]. Stone and colleagues (16) assessed perceptions of aging with the Attitude Towards Own Aging Sub-Scale [ATOA; (113)], and the Stigma Consciousness Questionnaire [SCQ; (114, 115)].

#### Injury analysis

Two studies solely addressed curling-related injuries placing this subtopic as a secondary finding (13, 14). Both acquired a reportive methodology to assess information on the demographics, site of injury, type of injury, and curling-specific aggravated conditions. A supplemental article (cross-referenced from delivery mechanics) addressed injury in terms of biomechanical delivery measurements according to force vector analysis of delivery positions (12). Studies concluded that curling is a safe winter sport compared to its Winter Olympic sports counterparts (13, 14).

#### Curling arena infrastructure

Another secondary finding was the subtopic of “Curling arena infrastructure” which contained two studies that discussed the construction and transformation of non-curling fields into curling ice rinks (10, 11). A novel ice arena and the National Aquatics Center located in China were venues prefabricated into curling arenas with curling ice installation (10, 11). These venues were analyzed to determine how the requirements of the curling ice and air differ from those of other arenas as well as traditional curling rinks (11). They reviewed the procedures and tests applicable to the design and operation of ice competition surfaces. Factors considered include the refrigeration load, graded static load, dynamic tests, vertical and horizontal deformation characteristics, and analytical frequency of buildings as a method of determining the infrastructure's effects on the playing surface and the development of a novel curling ice rink using detachable and prefabricated structures (10, 11).

#### Assessment of curling training and ability

The final secondary finding subtopic evaluated curling ability and training tactics. Two studies were assessed (8, 9). The primary assessment of curling ability was on-ice technical, strategic, and shot-making skills (8, 9). This constituted proficiency in accurately delivering the stone to the target, proficiency in producing desired shot outcomes for various types of shots, and a multiple-choice strategy test (8, 9). Secondary assessment of curling ability was determined via off-ice athletic ability by measuring curlers' muscular endurance, trunk strength, aerobic capacity, and flexibility, correlating the physiological tests to on-ice performance (8). Studies published prior to the completion of the literature search, had not made any consideration of gender differences within the sport of curling with the exception of sweeping and delivery mechanics (4, 62, 64).

## Discussion

This is the first scoping review aimed at defining and evaluating the quantitative knowledge that exists in the sport of curling. It was conducted through standard methods outlined by Arksey and O'Malley (5) to identify, select, and synthesize the findings from 94 studies. The current knowledge of the sport was documented by analyzing the geographic scope of studies, year of publication, and by the specific subdivisions of themes that make up the sport. Provided below are important results of this review that can be relevant for future researchers, coaches, and curling athletes.

The results from the included quantitative studies revealed substantial evidence of the controversy that exists in the curl mechanisms of the curling stone. To date, no unanimous theory exists to explain the lateral displacement of the curling stone (72, 75). Various physical models present conflicting proposals including: (1) the “front-back” and “right-left asymmetrical models” (2, 74, 75, 81, 97, 98); (2) the “evaporation-abrasion model” (86, 92, 96); (3) the “snowplow model” (81, 92); (4) the “pressure difference model” (92); (5) the “scratch-guide model” (73, 75, 89, 90); (6) the “pivot-slide model” (79, 85, 88); (7) the “split friction model” (87); and (8) the “water layer model” (76–78, 91–93). There is also a large variation in the different mathematical equations used in analysis across studies, as currently no numerical model of curling stone dynamics can satisfactorily predict all observed stone translations (80). Further controversy remains on the influence of the stone running band (72), speed of rotation (76, 87), and pebbled ice surface on the lateral displacement. Many studies have considered this domain of the sport, but few have observed the mechanisms experimentally or been able to provide concrete data. More research is needed in this area to develop statistical and relevant data for curlers, physicists, and ice makers to broaden comprehension of stone trajectories.

In addition, controversy still remains regarding the mechanisms of sweeping. Some studies support the thermodynamic theory, proposing that increased ice surface temperature produced through a combination of force and stroke speed lowers the coefficient of friction between the stone and ice (65, 68). Others support the scratch mechanism of sweeping, proposing that the combination of sweeping force and sweeping rate manipulates rock trajectory through the production of topographic scratches as opposed to surface heat (61). More research is needed to determine what influence each theory contributes to manipulating the longitudinal and lateral trajectory of the rock down the sheet of ice.

Technology is becoming a substantial factor in curling and its training tactics. The integration of AI agents and devices into curling formed its own theme in the results section, as the second most common focus. Across all variables of curling, AI programming has been most developed in terms of fabricating agents to learn and play the game, developing devices to enhance on-ice technical training of athletes, as well as for efficiency in statistical analysis and prediction of strategic curling outcomes (22, 23, 31–38, 43). Many studies with a focus on tactics and AI introduced computer algorithms into the continuous action spaces as a method of improving shot selection in curling. Technological devices were also present across sweeping studies, used to track force and acceleration measurements of the sweep stroke (64–67), as well as muscle activity of sweepers (4), and ice profiles after sweeping (61). Delivery mechanics literature further introduced technological devices. Online programs were utilized in terms of measuring kinematic motion (58), pressure and force vectors (12), as well as the stone position relative to the line of delivery (60). From the findings that robotic agents can outperform human curlers in game scenarios, we can accurately store digital curling informatics, and can greatly enhance accuracy in the training and testing of elite-level curlers. It appears that AI devices are an important factor in progressing the game and should continue to be developed, tested, and implemented in more areas specific to the sport of curling. Future directions in technological curling research are endless and should further investigate quantitative motions of the players and the stone according to specific game states by developing more adaptable IMU sensors. Another future direction is to develop instrumented “hacks” with built-in force plates that automatically generate feedback on the force produced. The “hack” is what curlers kick off of with their feet to begin sliding down the ice. This technological advancement could be used as a method of training memory in which curlers can learn kick speed and engagement of leg muscles in their takeoff of delivery for varying called weights.

Another important aspect observed from our review is the comparison across gender over the multifactorial variables of the game. Differences across gender were considered in terms of sweeping and delivery mechanics. Men and women were compared with respect to muscle activity and force output when sweeping, as well as their sweeping effect on the lateral and longitudinal trajectory of the stone (4, 62, 64). Studies across the variable of sweeping concluded that both genders produce similar muscle activation patterns when sweeping and that men have a more significant sweeping impact, carrying the rock a further distance and extending the stone's deceleration (4, 62). Differences in delivery mechanics between men and women were also of concern to some studies. The main finding established that women have greater hip flexibility and extension in a slide than their male counterparts (54). Gender comparisons failed to be considered in the evaluation of on-ice strategy calls, precision in shot-making ability, and psychological responses during gameplay. Future studies should consider expanding the differences according to gender in these game variables in order to develop a more comprehensive understanding of the difference between men's and women's play.

Substantial evidence of the differences between elite and sub-elite curling athletes was common across some themes. This comparison was most prevalent in studies regarding delivery biomechanics, in which elite and sub-elite curlers were contrasted in terms of core strength, flexibility, and balance with elite curlers outperforming their sub-elite counterparts (57). Comparisons also occurred for the center of mass, plantar pressure, and joint angles of elite and sub-elite curling deliveries (12). Timing methods used to estimate rock displacement were established to be reliable for elite athletes to vary stone delivery velocity, but not for sub-elite (56). Finally, skill level was compared according to attentional demands between sub-elite and elite curlers, showing that reaction time is distributed similarly across all skill levels, with elite-level curlers producing a slower delivery time overall (55). Comparison between skill levels was also assessed in sweeping; comparing force applied vertically, with elite athletes outperforming sub-elite (67).

There was a substantial difference in the magnitude of studies composed on the traditional format of curling as opposed to wheelchair curling. Wheelchair curling was only considered a subset of the sport, occupying six of the ninety-two included studies. Wheelchair studies primarily covered topics of the biomechanics of stone delivery (51, 52, 116), body composition of wheelchair curling athletes (50), and psychological skill training (20). It would be important to expand wheelchair curling knowledge to other areas of the game (ex, strategy variations from the traditional format), in order to optimize athlete development and performance by understanding variables specific to this format.

Although many aspects of sweeping were assessed through the literature, there is a dearth of current research assessing widely debated sweeping strategies. Corner sweeping, snow plowing, backward sweeping, and horizontal vs. vertical broom head stroke angles (Table 2) are all hotly debated concepts within the current curling world and clarification of their impact on rock trajectory needs to emerge via research.

**Table 2:** Newly debated sweeping techniques.

Sweeping Model	Diagram	Description
Corner sweeping	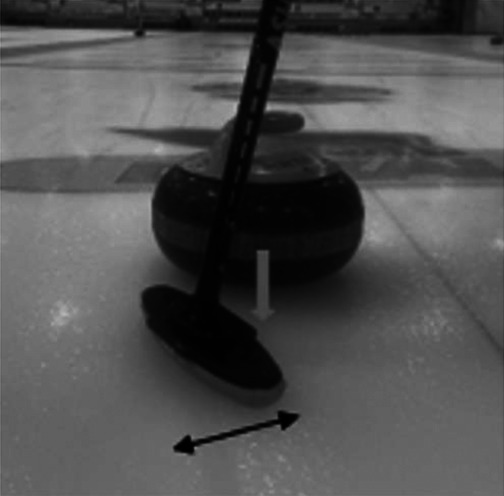	Locating your brush strokes at one side of the running surface rather than sweeping across the entire running surface.
Snow plowing	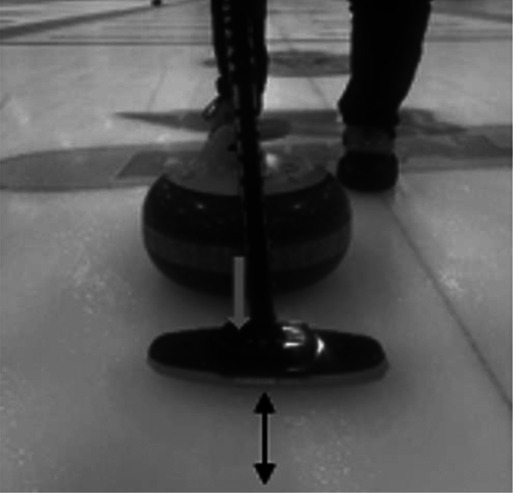	Producing brush strokes completely parallel to the forward motion of the stone. A method of building up debris on the broom. Then, when the broom is lifted, the rock's trajectory may be altered due to debris left in its path.
		Often called out as a rule violation in competitive play.
Backward sweeping	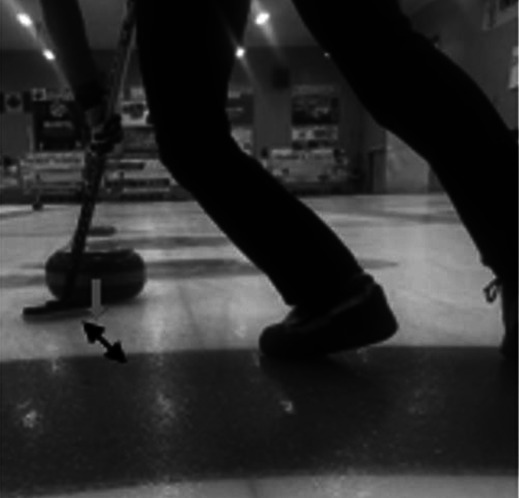	The distal sweeper positions their body to face the throwing hacks while simultaneously moving backwards, towards the far house in play. This establishes a brush stoke angle that matches the angle of topographic scratches produced by the proximal sweeper, who uses corner sweeping.
Horizontal vs vertical broom head angle	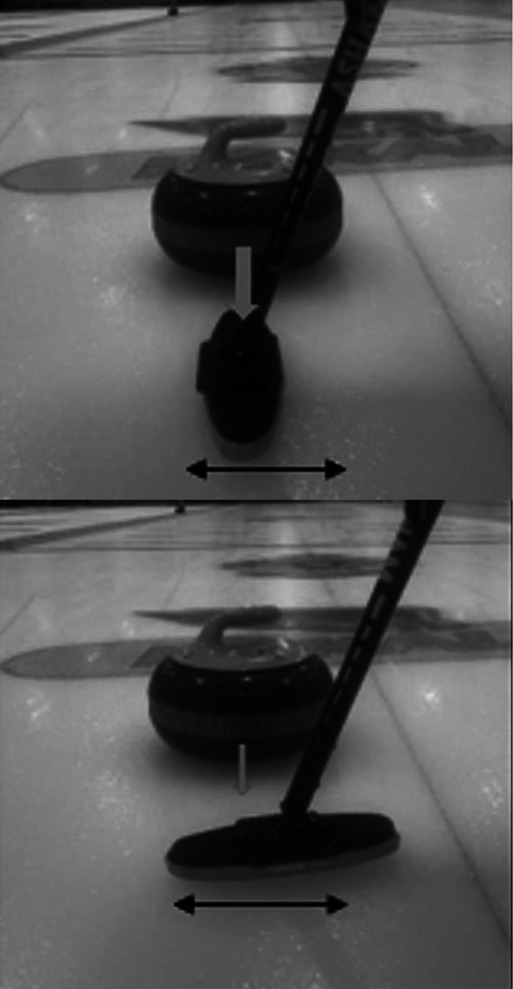	Horizontal broom head angle occurs when the wide head position moves perpendicular to the thrown rock. Vertical broom head angle occurs when the narrow head position moves perpendicular to the thrown rock


The black arrow depicts the motion of the broom head. The grey arrow depicts the motion of the rock.

### Future research

We feel that each variable discussed has relevance to curling athletes and/or future curling studies. There is undeniable value in understanding the physical mechanisms responsible for the generation of curl in a thrown stone; this game variable is prioritized as both a foundation of knowledge for the sport and a scientific variable of scholarly interest. Continued research may lead to new breakthroughs and continued advancement in how curling is conducted. There is also value in placing more emphasis on adaptable variables (e.g., technique, equipment, the focus of attention, rotation, etc.). Knowledge of game variables that an athlete has control over will be impactful to improving curling performance. Therefore, we believe future attention should be directed toward applied aspects of the game while continuing to seek concrete evidence for the underlying mechanisms.

It is notable that investigations of the mixed-doubles format of curling are absent from the extant literature. This novel format of curling was introduced in the Olympic Games in 2018 (117), was absent from all collected literature. With the variations in the rules, strategy, the number of players, gender, and length of the game, this format of curling requires attention in the research domain in order to develop a statistical understanding of its unique quantitative variables.

### Strengths and limitations of this scoping review

This review applied a systematic and rigorous search strategy that retrieved many articles to answer the research question. Each sub-factor in the sport of curling was searched with eight key search terms established in order to capture the depth of curling literature. However, due to limited access to study retrieval by the head researcher, some studies were unintentionally omitted and labeled as missing. In producing the review, both published journal articles and also grey literature (i.e., published thesis' and/or conference presentations) were considered. The consideration of grey literature, which met inclusion criteria, prevented the restriction of findings and served as a method of including all cases relevant to this sport. Further, the inclusion of thesis dissertations were a reliable source for collecting older variations of published articles that otherwise were unobtainable due to access restrictions. Several other relevant published studies written in other languages were omitted based on criteria precluding all but English; thus, it is important to point out that this may skew the geographic analysis of studies in favor of North America and Europe and limit accumulated global knowledge.

## Conclusion

This study sought to review the literature on the quantitative variables present in the sport of curling. It answers our research question and illustrates the current knowledge that exists in the sport for each devised variable, providing openings for future research. This review will aid in building a more comprehensive understanding of the game mechanisms, as well as add significantly to the understanding of the modern game techniques being applied to optimize athletes' gameplay. There is an influx of research being generated within the scope of curling in the most recent years, with research being conducted globally. The current body of literature reviewed reveals varying mechanisms underlying the curl trajectory of the stone and optimal dynamics of sweeping. The varying proposed explanations of the included studies are proof that more rigorous studies are needed to demonstrate the complex physics of the game. This review provides an impetus for further assessment on the variables of curling delivery biomechanics, wheelchair curling, psychological and strategic variables, and technology and tools. Studies included in this scoping review only scratched the surface of these variables and their impact on game performance.
